# Alveolar Abscess, with Caries or Necrosis—Their Phenomena, Treatment, &c, &c.

**Published:** 1870-06

**Authors:** H. L. Sage

**Affiliations:** Bridgeport, Connecticut


THE
### DENTAL REGISTER.

VOL. XXIV.] JUNE, 1870. [No. 6.

### Original Communications.

### ALVEOLAR ABSCESS, WITH CARIES OR NECROSIS—THEIR PHENOMENA, TREATMENT, &c., &c.

BY H. L. SAGE, BRIDGEPORT, CONNECTICUT.

In a paper presented for public perusal, and of course subject to criticism, it may be considered a merit that it be "short and pithy," to use a familiar phrase. But this will, I fear, be none too short; will suffer from the comparison as regards its brevity. It may be pithy, but not in the usual acceptation of the term. It may be dry, and in that respect partake of a natural characteristic of the substance denominated pith. If, in the treatment of my subject, I err by a prodigality of matter, so far as bulk is concerned, and it is wanting in the requisite "condensation, cogency, closeness and vigor of thought and style," charity would seem to suggest that the ability to develop these qualities in writing does not dwell with every one, and is a gift as rare as it is precious.

Having thus prefaced my subject, I would say that though the lesion termed alveolar abscess is not unfrequently forced upon the attention of the Dentist, and he attempts its treatment, with a view of restoring to health, so far as may be, the organs and tissues involved in disease, and the reproduction of lost portions; yet the various metamorphoses concerned in the processes of repair are none too well understood, and the ability to diagnose the true state of the parts during the transition from morbid conditions to conditions of health, and *vice versa*, as relating to the development or degeneration of the newly thrown out reparative material; in short, to distinguish the various appearances presented, are attainments comparatively limited, for the practice of sacrificing organs originating or exciting the trouble, obtains in by far the greatest number of cases.

Whether this arises from the disinclination of the patient to permit or, if commenced, to continue the necessary treatment, or the reluctance of the Dentist to assume the care or responsibility of cases the treatment of which are exceptions to the ordinary routine of practice, it matters not.

Then, too, the just when and what to do, how much and how often, and none the less important, how little, are points about which much may yet be learned, as regards the application of remedies, which may be made the means of good or evil, stimulative or destructive, depending upon the manner of their use, the idiosyncrasies of the patient, the local conditions presented, &c., &c , the main object being to aid and not retard the forces of nature in a change from a degenerative or disintegrative process to one reparative or building up.

An abscess in the alveolus may be acute or chronic; the latter, when of long continuance, or having been formed without the usual acute inflammatory symptoms, or so slowly or indolently as not to be observable by outward indications, or the amount of suffering attendant upon the formation of an acute abscess, which phenomena are in contradistinction to those manifested in the latter.

An alveolar abscess, more especially of the chronic class, always produces more or less absorption, disintegration or wasting of the osseous structures immediately adjacent to the inflamed periosteum. In cases comparatively rare, caries or necrosis of the maxilla becomes complicated with it; but such are not frequent, when taking into consideration the large number of abscesses which remain for years without producing these more serious results. When of long standing, or the above conditions present, their successful treatment becomes more difficult than in those designated as acute.

Alveolar abscess is usually excited by peridental inflammation, which is dependent upon causes familiar to all, though directly induced by irritation, whether that be produced by a blow on the tooth, a congested pulp or similar exciting agencies, perhaps conjoined with constitutional tendencies.

"We can not imagine," says Virchow, "inflammation to take place without an irritating stimulus (irritament). If, therefore, we speak of an inflammatory stimulus (irritament), we can not properly intend to attach any other meaning than that, in consequence of some change or other external to the part which falls into a state of irritation, and acting upon it either directly or through the medium of the blood, the composition and constitution of this part undergo alterations which at the same time alter its relation to the neighboring parts (whether they be blood vessels or other structures), and enable it to attract to itself and absorb from them a larger quantity of matter than usual, and to transform it according to circumstances. It may be assumed that it begins as an inflammation from the moment that the increased absorption of matter takes place."

In the case of a common abscess, its formation is attended by an increased infiltration of lymph (or lymph corpuscles), into a limited portion of tissue, causing induration and swelling. If its absorption takes place, and the formation of pus is thus prevented, it terminates in what is called *resolution.*

When the inflammation is not reduced, the nutrition of the part is interfered with to such an extent by the prevention of the normal circulation of the blood, in other words, by inducing stasis of the fluids, and thus rendering them unfit for purposes of nutrition, that degeneration or death ensues in a greater or less degree, according to the relative distance of the lymph from the peripheral portions of the infiltrated mass of tissue. The degeneration into pus of the central portions takes place because situated furthest from the sources of nutrition, and because the thickened and indurated intervening substance presents a barrier to the imbibition of nutrient fluids. In the peripheral portions, where the circulation is not so much obstructed, the lymph becomes more highly organized, and less so nearer the central. It usually presents both the fibrinous and corpuscular variety, and the indurated condition of the tissues in which the inflammation is seated is probably due more to the fibrin effused into them than to the presence of lymph corpuscles. The pus, which is continually forming and discharging, is surrounded by a wall of this hardened and expanded tissue ; so that the phenomena of what is usually denominated adhesive and suppurative inflammation, continues to be maintained by a fresh deposit of lymph on the one hand, and the degeneration into pus on the other, of that constituting the inner portions of the abscess, so long as inflammation continues, or until a change is produced in the local or constitutional conditions, one or both, upon which its production depended.

In regard to the formation of pus cells in tissues, in which lymph is abnormally exuded, the observations of Prof. Virchow and others of his school, go to show that they may be produced by changes in the nuclei peculiar to the textures themselves, and that in a deep-seated part changes in the cellular elements of the connective tissues of that part precede and accompany the formation of pus cells, by the elongation, division and multiplication of the nuclei, which ultimately assume the appearance of pus corpuscles, and that this multiplication or proliferation of the nuclei is the cause of the tense and hardened state of the tissue in the early stages of inflammation, and its subsequent softening is due to the separation of the nuclei from each other, which are changed into pus cells by an intercellular fluid substance.

One of the effects of inflammation being the effusion of coagulable lymph, this action may be induced in the periosteum of a tooth which has become affected, and if the inflammation be not removed, the process will continue.

The lymph exuded into or by the inflamed periosteum, will cause its thickening and ultimate detachment from the apex of the root, which thus forms a sac. The lymph by degeneration forms pus, and this, by distension of the sac, produces pressure against the surrounding alveolus, and ultimate absorption or disintegration of its walls, until a sinus opens externally for the passage of the pent-up and disorganized tissues, and the degenerated products of the inflammatory process, which will continue to form and be discharged until the "irritament" be removed, or such a change effected in its condition as to render its presence tolerable.

The only condition of cure recognized by most, has been the removal of the primary cause—the affected tooth—and it is not long since most writers on Dental Surgery advised such a course.

When caries or necrosis of the maxilla is present, but few, even in our own profession, would attempt the salvation of the tooth, especially when the destruction of the parts are considerable.

In a case reported by Dr. J. D. White, in 1865, in the *Cosmos*, vol. 6, page 530, of caries and necrosis of the maxilla over the right superior lateral incisor, the removal of the affected tooth was advised, and "reluctantly consented to," as a preliminary operation. A portion of the palatine process of the right maxillary bone, and the bottom of the socket, extending to the floor of the anterior nares, was necrosed.

That teeth have been saved in cases as unfavorable as the above, there is reason to believe, taking for granted the truth of the statements of men eminent in the profession.

The salvation of the tooth, and the restoration of the parts to health, including the reproduction of tissue in place of the destroyed portions of alveolus or maxilla, by the formation of what Virchow calls "osteoid tissue"—tissue which by taking up the calcareous salts becomes bone; in other words, "soft, uncalcified, osseous tissue," or as it may be stated, the ossification of newly developed fibrous tissue, or in other instances, of a substance similar in appearance to cartilage, and formed out of the products of inflammation—is now thought to be the object attainable, at least in many cases.

When the retention of the affected tooth is desirable and advisable, the object should be to effect such a change that the relation it sustains as an irritament may cease. This may be compassed by the application of medicinal agents, which, destroying the pus enveloping (shall I say pus-secreting?) membrane or sac, may also, by penetrating and uniting with the substance of the tooth root, remain to prevent by their antiseptic properties the future occurrence of disease in the structures which may be developed about it, so far as the irritation proceeding from the presence of a foreign body is concerned, at the same time changing the character of the inflammatory exudations, preventing their degeneration into pus, and assisting in their organization and development by exciting to healthy action, or as it has been stated, to "change the pus-producing to a plasm-producing surface."

But no cure can usually be expected by the use of medicaments alone, if carious bone is present. In the first stage only, which is inflammatory, are they effectual; Erichsen informs us, and Garretson recognizes the necessity of surgical interference, excepting in rare cases.

What is caries, as applied to bone? According to a common definition, "caries of bone is analogous to ulceration in the soft parts." Erichsen defines it as "the granular disintegration, and molecular death of the osseous tissue, with suppuration of the surrounding healthy parts." While necrosis is, in contradistinction, "the death of the bone as a whole."

The cancellous structures of bone are the most likely to be attacked by caries; the compact, by necrosis.

How may carious bone be distinguished? First, by the touch. Bare and rough, it seems soft and disintegrated, being easily punctured, and breaking down readily under the instrument; while in cutting away, small granules will be detached.

Healthy bone will be found the reverse; that is, hard, springy, resisting. Dr. J. D. White, in a report of the case before alluded to, speaks of the *dead* bone as being "very hard and firm."

Denuded bone is not necessarily indicative of caries.

Carious bone may be known, secondly, by the sight.

Its color may be grey, brown or blackish—dark, or dead-white, or oleagenous. In health, white and vascular, it will, if dead, be non-vascular, or destitute of blood vessels. This is the substance of the description given by the best authorities.

In what does the surgical treatment consist?

I reply (reference being had to its complication with alveolar abscess), in cutting or scraping away all dead portions, after obtaining sufficient room by the enlargement of the sinus. To effect this, cut, and then introduce a tent of cotton saturated with creosote or the tinc. of iodine, allowing it to remain a day or two, renewing it, if necessary, until of sufficient size to operate handily—care being taken not to render it larger than would suffice for the purpose.

The form of instruments suitable will suggest themselves to the operator. One of the most convenient in my experience has been a hoe, round or oval in shape, with an edge continuous with at least three-quarters of its circumference, and made in sizes to suit the chasm. In compact bone, as the alveolar or palatine plates, if perforated by caries, the edges may, perhaps, be better smoothed off by means of small rasps or files, and instruments with straighter edges than the round hoe—but when little room can be obtained, and the cavern is deep, or extending into the cancellous structures, the latter is preferable—in fact, the former would be almost or quite useless. Hatchets sometimes work well on the margins of plates. The bur drill of the Dentist, being similar in construction to the gouge recommended by Erichsen, and acting on the same principle, may be made occasionally useful. I obtained some very fine Palmer burs at the Dental Depot of S. S. White, which are much deeper and more delicately cut and sharper than those for ordinary use, and can speak of them as being very efficient for the purpose above stated.

If the roots of teeth are partially exposed and protruding into the opening produced by the abscess, they should be rounded off, polished, or left as smooth as possible, and filled solidly with gold. This can be very nicely and conveniently done after the inflammation produced by the operation has subsided, for the view of the apex of the root obtained by the opening makes it much more convenient than would otherwise be possible to determine when the pulp, or nerve canal is properly filled, besides preventing all danger of leaving particles of gold in the chasm, as is sometimes done in cases of less extensive disease. It may be necessary, in caries of the maxilla, to cut away several times before all the dead bone is removed, as on account of the comparatively small orifice which would ordinarily be obtained, a good view of the bone is somewhat difficult to secure, or very much so, when the teeth are retained.

After proper treatment, if the abscess does not become healthy, but pointed in shape, with one, or perhaps more, sinuses, leading off from certain locations in the cavity to certain portions of the bone, the operation of cutting or scraping must be repeated, until the affected osseous structures are all removed, which may be known, if proper treatment is instituted—for the walls of the abscess cavity will throw out healthy granulations, and lose their pointed and cone-like shape by the closure or filling up of the interior sinuses. A frequent use of the syringe during the operation is of course indispensable. The treatment of necrosis would seem to be more simple than that of caries, and its presence more apparent. The orthodox treatment is, to await the detachment of the sequestrum by the natural process, meanwhile keeping the parts as clean as may be by the free use of the syringe, loaded with suitable disinfecting and cleansing washes, and supporting the system by tonics, appropriate diet, &c.

Though it is not the practice to attempt the removal of the necrosed portion until its separation is complete, yet, Prof. Atkinson, if I mistake not, does not think it necessary to await the slow action of nature, but he advises, if proper treatment fails to resuscitate the bone, its removal, by cutting off all that portion deprived of circulation. (See paper on the "Reproduction of the Maxillary Bones," in the *Cosmos*, vol. 10, page 501, and read before the New York Medical Journal Association). The carious or necrosed bone having been removed, the reproduction of the lost structure is that to which reference must be had in the treatment.

When the reparative process has been established, an abscess of this kind does not differ essentially from an open wound or an ulcer, as regards the mode of repair, excepting, perhaps, in the later stages of development into bone.

There will not be healing by what Paget calls *primary adhesion*, Hunter, *adhesive inflammation* ; for in order to effect this, the surfaces must come into contact after the lymph, plastic matter, or plasm, has been thrown out by the inflammatory process, and if it is possible that it be accomplished by this mode, without the close approximation of surfaces, the coagulable lymph, plasm, or fibrin must be protected and held undisturbed in the pocket or matrix during its upward progress into fibro-cellular or connective tissue.

It is difficult to afford this protection when the cavity of the abscess, and the sinus leading thereto, have been much enlarged by an operation for caries or necrosis, thus permitting easy egress to the inflammatory exudation, or rendering it liable to injury by contamination with fluids or substances foreign to the chasm.

The mode of repair will usually be limited in the first instance to the healing by granulations, and in the second by the union of granulations or secondary adhesion, when the opposing surfaces come into contact by the continuous exudation, organization and development of lymph.

"Granulations form in depth," says Paget, "from one to three lines." If it is a supposable case, which can hardly be admitted, we will take an open alveolar abscess, with a cavern produced by the destruction of the maxilla and the soft parts, of say seven lines in diameter. If only three lines at the most is the depth of a cicatrix, is the chance remote, to say the least, of filling up this chasm, even allowing for the contraction of tissue? Would not the location of the abscess in the jaw, and the ultimate development of the newly-formed fibro-cellular, or osteoid tissue into bone, render it more favorable than if entirely in the soft parts, for the continuous formation and development of granulations until the lost tissues were all reproduced, though in a less perfect form than before.

Does the rule hold good both as regards the restoration of bone and the soft parts? Such a conclusion is, I think, not warranted. The reparative material for the closure of the fistula of the abscess forms a fibro-cellular tissue, and likewise for the reproduction of the alveolus; but in the latter may go a step further, and by the absorption of the lime salts, or partaking of the natural products of the vessels of the part, become ossified.

If the development of granulation cells into tissue outstrips the destructive process, induced by the endeavor to keep up an amount of inflammation on the surface sufficient to cause the exudation of granulation substance, letting the inflammation subside sufficiently for its development, and again inducing inflammation when necessary to promote a fresh exudation, will the depth of a cicatrix exceed three lines on the circumference of a chasm in which a greater depth is required, for its closure in upon itself, in order to completely fill the space made vacant by the destructive process?

This may not seem unimportant when it is desirable to restore the parts fully, or nearly so, in order to save the teeth involved. The walls of the abscess, of course, approach each other from every side; but this would not suffice for a full restoration in a cavity seven or more lines in diameter, if the rule in regard to the maximum depth usually reached, in the formation of granulations, applied to the bone developing tissue. But it does not.

Would it apply if the newly formed tissues—so far as bone formerly extended—did not develop into bone? I think not.

However, I do not suppose that a cavity could be produced in the jaw of seven lines in diameter, from all surfaces, taking the usual conformation, and hardly, if we included both the destroyed soft and osseous structures.

The above supposition is brought forward only for an illustration, not that it is likely that such a case could be presented, excepting in the mouth of a giant, or that of an animal other than human; though I have under treatment a case in which one portion of the chasm reached a depth of six lines, and in opposite direction exceeded that, the successful treatment of which, with the retention of the teeth included, I hope to be able to report at a future day.

In the treatment of an abscess in which the destruction of tissue has not been great, after suppurative inflammation has been checked, and thus the development of lymph in adhesive inflammation promoted, the lips of the orifice will close by this latter process. The filling up of the inside of the abscess cavity will also be accomplished in like manner—that is, by the production of a fibrinous material—while an abscess in which the loss of parts has been extensive, will heal from the bottom and sides by the development into fibres of cells with intercellular substance ; in other words, granulations. In the former mode of healing, the fibrinous variety of lymph is the preponderating reparative material ; in the latter, the corpuscular ; so that the degree in which lymph is favorable or unfavorable for development is greatly influenced by, or dependent upon, the character of the inflammatory exudation, or in relative proportion to the presence of the fibrinous or corpuscular elements, and the latter being the most unfavorable, we may infer that the mode of repair by granulations is a more morbid phenomena than that by the adhesive inflammation. Previous to the formation of granulations, a fluid resembling the white blood corpuscles deposited in a fibrinous film, is exuded on the surface. This produces a whitish substance (glazing), which increases in thickness for from one to ten days, according to the character of the textures in which the abscess or wound may be located.

In bone, when cut or scraped, the maximum period may be required for the commencement of a change in this stage of the phenomena, when compact in structure, but if cancellous, the transition stage is shorter ; while if in the soft parts, about three days is sufficient. Then follows an increased determination of blood to the parts to be repaired ; next the exudation of material to be organized into granulations, which Hunter describes as having to the naked eye the appearance of coagulable lymph, and which soon assumes vascularity.*

Granulations, in appearance, Dungleson describes as "reddish, conical, flesh-like shoots." Paget informs us that they have a "bright, ruddy texture, a granulated free surface, are succulent, and abundantly supplied with blood." They are composed of cells, arranged without any apparent order,

---

*Paget's Surgical Pathology.

which develop into fibro-cellular or connective tissue, or degenerate into pus cells.

Paget affirms that " superficial cells are detached in pus while deeper ones seem to be developing into filaments." Pus is always produced on an exposed granulating surface.

An application of the chloride of zinc effectually prevents this for a period of from three to five or more days, according to the strength of the solution. Its effect is to form a coagulum or eschar, which remains to prevent the irritating effects of air and moisture upon the raparative material, and its taint by outside fluids, and the walls of the abscess will need no other dressing so long as the eschar remains united thereto. Other local medicaments stimulating in their nature, and which will not at the same time produce too much inflammation, thus preventing the development of the granulating cells into tissue, are useful, if applied of the requisite degree of strength and at proper periods—for there is a certain point in the intensity of the inflammation which such an agent, in addition to natural causes, may produce, to go beyond which is fatal to all development, so long as it continues.

Such an agent may excite it in an undue measure by overstimulation, if used in too great strength or too often, and thus defeat the object for which it is applied. To stimulate, but not destroy, in the periods of repair, after the cause of irritation is removed, or by a change ceases to excite it, would usually be considered desirable; or if an eschar be formed by the superficial destruction of the walls of the abscess, in order to protect the parts beneath, some respite must then be had in order to give the latter time to develop. If the escharotic or coagulum-forming agents, such as the chloride of zinc in strong solution, be not employed, but milder dressings are indicated, antiseptic and stimulant, a weak solution may be useful. A mild dressing of the tincture of iodine, as a stimulant, is very good for daily use, though the chlo. of zinc solution may be made of suitable strength for frequent application, and acts very satisfactorily.

When the use of the syringe is indicated, an excellent, mild, antiseptic and stimulating wash, which may be safely applied by the patient at proper intervals between the sittings, when such cleansing is called for, and the sinus is large enough to permit it, is one the formula for which I here produce, and which may be found on page 67, of *White's Materia Medica.*

It is as follows:

℞ Tinct. iodinii comp., M. XIV.; acid carbolic chryst., m. VI.; glycerina, ℥ I; aq. destillat, ℥ V.

For a dressing on the gum, over the mouth of the fistula, a combination of tannin and glycerin is recommended. What part the tannin performs may be best learned by the following, from Erichsen: "Astringents directly applied to the inflamed parts are of extreme service in those forms of congestive or passive inflammation in which the circulation is sluggish and the capillaries loaded by inducing the contraction of the vessels."

From the *Dental Materia Medica* we also learn that "it unites with albumen, fibrin and gelatin, forming insoluble tannates, thus preserving the parts beneath from the influence of irritating agents." The glycerin is used as a solvent of and vehicle for the tannin. The partiality to this combination as an outward dressing for the abscess, that is, over the lips of the sinus, and to the chloride of zinc for the abscess proper, is not had without good reason. For checking the discharge or formation of pus, I have found nothing so effectual as the latter.

Other agents are often employed either alone or in combination with the old-fashioned remedy, the tincture of iodine, to meet certain indications, such as creosote or carbolic acid; but to all of which I do not propose to refer. Creosote has long been a favorite remedy in the direct treatment of alveolar abscess, and with its "stimulant, narcotic, irritant, styptic, antiseptic, caustic and escharotic qualities," may surely meet a variety of indications. It is an invaluable agent, and is, or has been, more frequently employed in simple alveolar abscess than any other known remedy, especially as a preliminary application for cauterizing the sac, &c.

Of the remedies for incipient abscess time will not permit me to speak; but there is one combination which, locally applied over the root of the affected tooth, is often of great service, as I have found it. It was suggested in the *Cosmos*, by Dr. Frank Abbott, and consists of the officinal tinct. of iodine and the tinct. of aconite root, in equal proportions. But the difficulty of knowing what to apply is not so great as that of knowing when and how, and to be able to judge of the actual state of the parts. It is quite necessary, or at least desirable, to be able to distinguish the products of inflammation as they exhibit favorable or unfavorable conditions, in the attempt to effect a restoration to health. Coagulable lymph, lymph corpuscles and pus being very frequently found together, or in succession, on the same surface, or in the same tissues, let me briefly refer to them. Their appearance, as usually described by different writers, may be thus summed up: Coagulable lymph is a substance, "colorless, trembling, gelatinous,"—"whitish, greyish, or yellowish-white; elastic, semi-transparent,"—"ropy or stringy to the touch, or between the fingers."

According to Prof. Gross, "recently effused lymph exhibits a whitish, pale straw or opaline appearance, is of a soft, unctious consistence, like hot glue or a thin solution of starch, but does not always present the same appearance." Chemical constitution, "fibrin in union with albumen." Lymph exuded in the inflammation of bone, and likely to ossify, Rokitansky describes as, "at first a dark red substance, gelatinous, and which gradually becomes white and firm in texture, becoming like soft, flexible cartilage, and then like ruddy succulent bone."

Plasm is defined by Prof. Atkinson as "healthy juices of flesh, brought to a fractured tissue for repair or nourishment;" and pus as "broken down plasm, consisting of dead white blood corpuscles, simply deteriorated plasm."

As white blood corpuscles are indistinguishable from lymph corpuscles, would it be difficult to decide whether or not corpuscular lymph and plasm are identical? Would it not be natural to suppose that, according to the above definition, they do not materially differ? Plasm would usually, in all probability, be a mixture of fibrinous lymph and lymph corpuscles.

Sanies, the Dr. says, "is disintegrated plasm and tissue in a state of chemical solution, on the downward scale," &c. In addition it might be called the degeneration, breaking up or division of the pus cells.

Pure pus, as usually described, is "thick or cream like, or yellowish; opaque, smooth and slightly glutinous to the touch." To deduce a few inferences from Paget and others: Pus cells well constructed and ill developed granulation cells, are not distinguishable from each other. Pus cells, inflammatory lymph and the material of granulations, are essentially similar; but the first, unlike the last, wholly fail of development, or if developed to a certain extent degenerate. Corpuscular lymph, granulation cells and the white blood corpuscles (lymph corpuscles?), in apparent structure do not differ. "To distinguish between pus cells and colorless blood cells," Virchow affirms, "is not possible, unless their home and mode of origin be known. If external to the blood, they will be found pus cells; if not, colorless blood cells." Lymph while it is the vehicle of certain substances from the tissues to the blood, likewise conveys the corpuscular elements, and an increase of blood in a part is a large increase of colorless cells; that is, lymph corpuscles. This non-coagulable portion of the lymph is capable of development, "by passing upward through fibro cells, caudate cells, &c., into filaments, or of degeneration into pus corpuscles, granule cells," &c.*

*Erichsen's Science and Art of Surgery.

To close this somewhat desultory paper, I will briefly state some of the conditions necessary for the production and development of lymph into tissue.

And first, for the production, an inflammatory process is necessary. If the abscess is indolent, it may be required to induce the exudation by artificial means. Secondly, for its development, if the fibrinous element preponderates, the chances are much greater than in lymph composed principally of corpuscles. "Rich in fibrin, and that clear homogeneous tough and filamentous," as Paget regards it, is eminently possessed of the essential qualities for a reparative material, though the two varieties are seldom found unmixed. Constitutional health and vigor, and blood rich in the life-giving and life-supporting elements, and the removal of the irritation or the prime cause of the lesion, are among the favorable conditions. Lastly, while the degree of inflammation regulates the supply of lymph, much inflammation producing the exudation of much lymph, and *vice versa;* yet the least extent to which the inflammatory process can be carried consistent with the effusion of the reparative material is the most favorable for its development; in other words, the less normal nutrition is interfered with, the more likely is the lymph to organize and assimilate in structure with the tissues, from whose vessels the lymph was derived. No development will be effected so long as the inflammation continues, according to Paget, or in the words of Erichsen, if it proceeds beyond a certain point in intensity. Just where that point is, is not known, I fancy, and only a vigilant and practiced eye can detect, by present and future indications, how nature may be aided or retarded in the work of restoration. To stimulate to an exudative process and to a process of development, are two different things. As a general thing, in simple alveolar abscess, "remove the original cause, and the effects will disappear," for nature will do all the work.

But the object is not always to remove, but to change the state or condition of the structures, and the relation one sustains as an irritant to the other. In short, to bring about such a compatibility between, or tolerance of, each other, that a divorce may be prevented and all breaches healed.

